# Dual-center development and validation of a LDL-C and TC based nomogram for preoperative hemorrhagic risk in adult moyamoya disease

**DOI:** 10.3389/fnut.2026.1846841

**Published:** 2026-05-25

**Authors:** Haitao Wu, Mingchen Xie, Tingxuan Wang, Luo Li, Jian Xu

**Affiliations:** 1 Department of Neurosurgery, The Affiliated Hospital of Qingdao University, Qingdao, Shandong, China; 2 Department of Neurosurgery, Qingdao Hospital, University of Health and Rehabilitation Sciences (Qingdao Municipal Hospital), Qingdao, Shandong, China

**Keywords:** dual-center risk prediction, low-density lipoprotein cholesterol, Moyamoya disease, preoperative intracranial hemorrhage, Total cholesterol

## Abstract

**Introduction:**

Moyamoya disease (MMD) is a chronic cerebrovascular disease characterized by progressive stenosis of intracranial arteries and compensatory formation of abnormal collateral vessels. Preoperative intracranial hemorrhage represents a severe and catastrophic complication in adult patients with MMD, while reliable strategies for precise risk stratification are still lacking to date. Furthermore, the potential association of lipid metabolic indices with preoperative hemorrhagic risk in MMD remains poorly clarified and insufficiently explored.

**Methods:**

A retrospective multicenter study was conducted enrolling 260 adult MMD patients recruited from two medical centers during 2015–2025. All participants were categorized into the hemorrhage group (*n* = 57) and non-hemorrhage group (*n* = 203). Univariate analysis, LASSO regression with 10-fold cross-validation, and multivariate logistic regression were sequentially performed to screen and determine independent risk factors. A predictive nomogram was further constructed, and its predictive performance was comprehensively validated via receiver operating characteristic (ROC) curve, calibration curve, decision curve analysis (DCA), and clinical impact curve (CIC).

**Results:**

Elevated LDL-C was confirmed as an independent risk factor for preoperative hemorrhage (OR = 2.46, 95%CI: 1.41–4.28, *p* = 0.002). Restricted cubic spline regression demonstrated a linearly positive correlation between LDL-C, TC and preoperative hemorrhagic risk, with optimal cut-off values of 3.5 mmol/L and 5.0 mmol/L, respectively. The ROC showed moderate discriminative value with an AUC of 0.78 (95%CI: 0.72–0.85), accompanied by satisfactory sensitivity, specificity and favorable clinical net benefit.

**Conclusion:**

Elevated LDL-C is independently correlated with the occurrence of preoperative hemorrhage in adult MMD patients. The nomogram based on LDL-C and TC serves as a concise and clinically practical tool for moderate-risk stratification of preoperative hemorrhage, which is conducive to facilitating individualized clinical management and therapeutic decision-making for MMD.

## 1 Introduction

Moyamoya disease (MMD) is an idiopathic chronic cerebrovascular disorder defined by progressive luminal stenosis of the bilateral internal carotid artery terminal segments—culminating in obliteration—and concurrent involvement of the proximal segments of the anterior and middle cerebral arteries; a defining pathological hallmark entails the *de novo* formation of dysplastic collateral vascular networks at the cranial base, which manifests the pathognomonic “moyamoya” (smoke-like) angiographic phenotype (1, 2). The pathogenesis of MMD remains multifactorial, incompletely elucidated, and inherently intricate, encompassing the sophisticated crosstalk among diverse etiopathogenic drivers—including dysregulated immune signaling cascades, local hemodynamic perturbations, and other incompletely characterized molecular and environmental determinants—that synergistically orchestrate vascular remodeling and occlusive vasculopathy (3, 4). MMD epidemiology is characterized by pronounced geographical and ethnic heterogeneity—a phenomenon reflected in substantial global variability in incidence across distinct geographical regions and ethnic cohorts. Contemporary epidemiological evidence demonstrates that annual incidence rates in Asian populations, particularly individuals of East Asian ancestry, range from 0.48 to 2.3 per 100,000 individuals, markedly exceeding the corresponding rates reported in European and North American populations (approximately 0.04 to 0.33 per 100,000) (5–7). With respect to age distribution, MMD exhibits a characteristic bimodal pattern, with two primary peaks occurring in childhood (around 5 years of age) and middle adulthood (around 40 years of age) (8–10).

Based on distinct clinical manifestations, Moyamoya disease can be stratified into two major subtypes: ischemic and hemorrhagic. The ischemic subtype predominates disproportionately in pediatric populations, characterized by prototypical presentations including transient ischemic attacks (TIAs), reversible hemiparesis, dysarthria, aphasia, and progressive cognitive deterioration (11). In contrast, the hemorrhagic subtype is more prevalent among adults, where intracranial hemorrhage—frequently involving the ventricular system, basal ganglia, and subarachnoid space—represents a life-threatening neurosurgical emergency of utmost urgency (12). Notably, intraventricular extension of intracerebral hemorrhage has been validated as a key determinant of poor early prognosis and elevated in-hospital mortality in acute hemorrhagic stroke, independently exacerbating neurological deterioration via secondary hydrocephalus and brainstem compression (13). As a general rule for spontaneous intracerebral hemorrhage (ICH), anatomical topography determines clinical phenotypes and early prognosis: lobar ICH exhibits distinct clinical features and a significantly worse early prognosis compared with deep subcortical ICH (14). Moreover, non-hypertensive pathogenic mechanisms of ICH—including cerebral amyloid angiopathy, vascular malformations and coagulation disorders—are predominantly associated with lobar brain involvement. Despite remarkable advancements in modern neuroimaging modalities—including high-resolution MRI/MRA and digital subtraction angiography (DSA)—that have substantially facilitated the early detection and characterization of MMD (15), the inherent risk of intracranial hemorrhage in adult patients prior to surgical intervention remains an intractable clinical challenge. This risk exerts a profound and enduring adverse impact on patients’ quality of life and long-term prognostic trajectories, often culminating in catastrophic neurological sequelae or fatal outcomes (16, 17). A study demonstrated that (12)36.7% of patients with hemorrhagic MMD developed recurrent hemorrhages, with an average annual incidence of 4.5%12. Of these, 19.1% succumbed to rebleeding, and 12 patients sustained severe disability. The cumulative rebleeding risk was 7.8% at 5 years, 22.6% at 10 years, and 35.9% at 15 years.

However, the current body of research on independent risk factors for preoperative hemorrhagic events in adult MMD patients remains scant. Therefore, this study aims to identify and validate independent risk factors for preoperative hemorrhage in adult patients with moyamoya disease by analyzing data including age, comorbidities, neuroimaging findings, and laboratory parameters. We also attempt to establish a simple and clinically practical predictive model based on these key factors.

Our findings may provide clinicians with reliable evidence for risk stratification and facilitate the formulation of personalized treatment regimens, which may help improve patient-centered therapeutic management.

## 2 Subjects and methods

### 2.1 Selection of clinical data

This was a retrospective analysis. Electronic medical records of patients diagnosed with MMD at Qingdao Municipal Hospital and the Affiliated Hospital of Qingdao University between January 2015 and January 2025 were reviewed. The study was approved by the Ethics Committee of Qingdao Municipal Hospital and the Affiliated Hospital of Qingdao University, and all procedures were performed in strict accordance with the ethical principles stated in the Declaration of Helsinki.

Inclusion criteria: (1) According to the guidelines issued by the Japan MMD Research Committee (2, 16), patients were diagnosed with MMD via digital subtraction angiography (DSA) or magnetic resonance angiography (MRA) (18). (2) Patients were aged > 18 years.

Exclusion criteria: (1) Patients with a history of concurrent brain tumors, cranial radiotherapy, Down syndrome, neurofibromatosis, meningitis, hyperthyroidism, systemic lupus erythematosus, or sickle cell disease; (2) Patients diagnosed with moyamoya syndrome; (3) Patients with incomplete clinical information (e.g., imaging data, biochemical indicators).

### 2.2 Collection of clinical data

This study collected basic demographic data from 260 patients, including sex, age, body mass index (BMI), history of hypertension, diabetes mellitus, coronary heart disease, hyperlipidemia, smoking, alcohol consumption, anticoagulant drug use, lipid-lowering drug use, family history of cerebrovascular disease, and lesion laterality (unilateral/bilateral) of moyamoya disease, as well as serum creatinine (Scr), staging of the anterior choroidal artery, staging of the posterior communicating artery, and Suzuki staging. The staging of the anterior choroidal artery grade (AChA grade), posterior communicating artery grade (PComA grade), and Suzuki staging were independently evaluated by at least two neurosurgeons based on imaging findings. At the same time, we also collected the hemoglobin (Hb), albumin, high-density lipoprotein cholesterol (HDL-c), low-density lipoprotein cholesterol (LDL-c), total cholesterol (TC), and triglycerides (TG) indicators of the patients at their first admission, for further analysis.

### 2.3 Diagnosis of preoperative intracerebral hemorrhage in MMD

Preoperative hemorrhage in MMD patients was strictly defined as acute or subacute spontaneous intracranial hemorrhage identified at the patient’s first clinical visit, confirmed by typical hemorrhagic symptoms and radiological evidence. This outcome refers exclusively to the initial hemorrhagic event at first presentation, and does not include remote old intracranial hemorrhage history occurring before surgery.

### 2.4 Statistical methods

Analytical procedures were performed using R software, GraphPad Prism, and DecisionLinnc software. Patients were stratified into two groups according to the presence or absence of preoperative intracerebral hemorrhage. Categorical variables were reported as frequencies and percentages, and between-group differences were analyzed using the chi-square test or Fisher’s exact test as appropriate; continuous variables were assessed for normality via the Kolmogorov–Smirnov test, with normally distributed data presented as mean ± standard deviation (SD) and compared using the independent-samples t-test, and non-normally distributed data expressed as median and interquartile range (IQR) and analyzed via the Mann–Whitney U test. Univariate binary logistic regression was initially conducted to screen candidate risk factors for preoperative hemorrhage. Subsequently, LASSO logistic regression with ten-fold cross-validation was applied to further identify robust core predictors from the candidate variables, which effectively mitigated model overfitting and multicollinearity and optimized variable selection stability. Variance inflation factor (VIF) was calculated to evaluate multicollinearity among screened variables. Multivariate binary logistic regression was then performed to determine independent risk factors for preoperative hemorrhage. Restricted cubic spline (RCS) models were adopted to flexibly explore the dose–response relationship between lipid indicators and hemorrhagic risk. The discriminative performance of the predictive model was assessed using receiver operating characteristic (ROC) curves and the area under the curve (AUC); internal validation and calibration were conducted using 1,000 bootstrap resamplings, and model goodness-of-fit was verified by the Hosmer-Lemeshow test. A nomogram was constructed to translate statistical findings into clinically applicable risk stratification. Finally, decision curve analysis (DCA) and clinical impact curve (CIC) were performed to comprehensively evaluate the clinical utility of the model. All statistical tests were two-sided, and *p* < 0.05 was considered statistically significant.

## 3 Results

### 3.1 Distribution of different characteristic variables in patients with moyamoya disease in the population and different types and comparison between groups

A total of 260 MMD patients were enrolled and stratified into the non-hemorrhagic group (*n* = 203) and hemorrhagic group (*n* = 57) based on the presence of preoperative intracranial hemorrhage (Table 1). Between-group comparisons of baseline characteristics demonstrated no significant differences in age, sex, diabetes mellitus, coronary heart disease, smoking history, alcohol consumption history, family history of cerebrovascular disease, hypertension, lesion laterality (unilateral/bilateral), AChA grade, PComA grade, serum creatinine (Scr), or anticoagulant drug use (all *p* > 0.05). In terms of disease staging, no significant intergroup difference was detected when stratified into Suzuki stages I–II and III–V (*p* = 0.171). Notably, significant between-group differences were observed in body mass index (BMI), prevalence of hyperlipidemia, and lipid-lowering drug use (all *p* < 0.05). For laboratory parameters, no significant intergroup differences were found in HDL-C, HGB, TP, ALB, or TG levels (all *p* > 0.05), whereas LDL-C and TC concentrations were significantly higher in the hemorrhagic group (both *p* < 0.001).

**Table 1:** Baseline characteristics of the included patients.

Variables	Total (*n* = 260)	Hemorrhagic MMD		*P*
		Absent (*n* = 203)	Present (*n* = 57)	
Age, Mean ± SD	47.43 ± 11.51	47.54 ± 11.28	47.05 ± 12.38	0.777
Sex, n(%)				0.464
Male	116 (44.62)	93 (45.81)	23 (40.35)	
Female	144 (55.38)	110 (54.19)	34 (59.65)	
Diabetes, *n* (%)				0.347
No	216 (83.08)	171 (84.24)	45 (78.95)	
Yes	44 (16.92)	32 (15.76)	12 (21.05)	
CHD, *n* (%)				0.622
No	239 (91.92)	188 (92.61)	51 (89.47)	
Yes	21 (8.08)	15 (7.39)	6 (10.53)	
Smoking history, *n* (%)				0.215
No	223 (85.77)	177 (87.19)	46 (80.70)	
Yes	37 (14.23)	26 (12.81)	11 (19.30)	
Alcohol consumption history, *n* (%)				0.892
No	245 (94.23)	192 (94.58)	53 (92.98)	
Yes	15 (5.77)	11 (5.42)	4 (7.02)	
Family history, *n* (%)				0.892
No	245 (94.23)	192 (94.58)	53 (92.98)	
Yes	15 (5.77)	11 (5.42)	4 (7.02)	
Side of MMD, *n* (%)				0.271
Unilateral	117 (45.00)	95 (46.80)	22 (38.60)	
Bilateral	143 (55.00)	108 (53.20)	35 (61.40)	
HTN, *n* (%)				0.950
No	177 (68.08)	138 (67.98)	39 (68.42)	
Yes	83 (31.92)	65 (32.02)	18 (31.58)	
Hyperlipidemia, *n* (%)				0.005
No	222 (85.38)	180 (88.67)	42 (73.68)	
Yes	38 (14.62)	23 (11.33)	15 (26.32)	
Hypolipidemic, *n* (%)				0.002
No	238 (91.54)	192 (94.58)	46 (80.70)	
Yes	22 (8.46)	11 (5.42)	11 (19.30)	
Anticoagulant drugs, *n* (%)				0.418
No	249 (95.77)	196 (96.55)	53 (92.98)	
Yes	11 (4.23)	7 (3.45)	4 (7.02)	
Suzuki stage, *n* (%)				0.170
I-II	179 (68.85)	144 (70.94)	35 (61.40)	
III-V	81 (31.15)	59 (29.06)	22 (38.60)	
AChA grade, *n* (%)				1.000
0	4 (1.54)	3 (1.48)	1 (1.75)	
1	92 (35.38)	72 (35.47)	20 (35.09)	
2	164 (63.08)	128 (63.05)	36 (63.16)	
PComA grade, *n* (%)				0.884
0–1	93 (35.77)	72 (35.47)	21 (36.84)	
2	167 (64.23)	131 (64.53)	36 (63.16)	
Scr	63.04 ± 19.74	63.64 ± 20.80	60.89 ± 15.36	0.354
BMI	23.60 ± 3.59	23.28 ± 3.74	24.74 ± 2.71	0.006
HDL, Mean ± SD	1.18 ± 0.34	1.16 ± 0.34	1.24 ± 0.32	0.097
LDL, Mean ± SD	2.60 ± 0.93	2.37 ± 0.71	3.40 ± 1.14	<0.001
TC, Mean ± SD	4.34 ± 1.15	4.08 ± 0.86	5.26 ± 1.53	<0.001
HGB, Mean ± SD	137.50 ± 15.77	138.20 ± 15.72	135.02 ± 15.86	0.179
TP, Mean ± SD	66.00 ± 6.83	65.78 ± 6.82	66.80 ± 6.88	0.320
ALB, Mean ± SD	36.42 ± 6.55	36.46 ± 8.81	36.29 ± 6.31	0.967
TG, Mean ± SD	1.41 ± 0.61	1.40 ± 0.59	1.44 ± 0.68	0.679

CHD, Coronary heart disease; HGB, Hemoglobin; TP, Total Protein; TG, Triglycerides; TC, Total Cholesterol; HDL, High-Density Lipoprotein; LDL, Low-Density Lipoprotein; AChA grade (anterior choroidal artery grade); PComA grade(posterior communicating artery grade).

### 3.2 Risk factor analysis and prediction model construction for preoperative hemorrhage

#### 3.2.1 Univariate and multivariate analysis of risk factors for preoperative bleeding in moyamoya disease

Using preoperative hemorrhage in MMD patients as the dependent variable, univariate and multivariate logistic regression analyses were sequentially performed for all baseline indicators. Univariate logistic regression showed that age, sex, diabetes mellitus, coronary heart disease, smoking history, alcohol consumption history, family history of cerebrovascular disease, hypertension, lesion laterality (unilateral/bilateral), AChA grade, PComA grade, HDL-C, HGB, TP, ALB, TG and anticoagulant drugs use were not significantly associated with preoperative hemorrhage risk (all *p* > 0.05); while hyperlipidemia, lipid-lowering drugs use, LDL-C and TC were significant risk factors for preoperative hemorrhage (hyperlipidemia: OR = 2.80, 95%CI: 1.34–5.81, *p* = 0.006; lipid-lowering drugs use: OR = 4.17, 95%CI: 1.70–10.22, *p* = 0.002; LDL-C: OR = 3.43, 95%CI: 2.32–5.08, *p* < 0.001; TC: OR = 2.58, 95%CI: 1.86–3.58, *p* < 0.001) (Table 2).

**Table 2:** Univariate and multivariate logistic regression results.

Variables	Univariate -*P*	OR	Multivariate -*P*	OR
Age	0.776	1.00 (0.97–1.02)		
Sex				
Male		1.00 (Reference)		
Female	0.464	1.25 (0.69–2.27)		
DM				
No		1.00 (Reference)		
Yes	0.348	1.43 (0.68–2.99)		
CHD				
No		1.00 (Reference)		
Yes	0.445	1.47 (0.54–3.99)		
Smoking history, *n*(%)				
No		1.00 (Reference)		
Yes	0.218	1.63 (0.75–3.54)		
Alcohol consumption history, *n*(%)				
No		1.00 (Reference)		
Yes	0.648	1.32 (0.40–4.30)		
Family history, *n*(%)				
No		1.00 (Reference)		
Yes	0.648	1.32 (0.40–4.30)		
Hyperlipidemia, *n*(%)				
No		1.00 (Reference)		
Yes	0.006	2.80 (1.34–5.81)		
Hypolipidemic, *n*(%)				
No		1.00 (Reference)		
Yes	0.002	4.17 (1.70–10.22)		
Anticoagulant drugs, *n*(%)				
No				
Yes	0.246	2.11 (0.60–7.49)		
Side of MMD				
Unilateral		1.00 (Reference)		
Bilateral	0.273	1.40 (0.77–2.55)		
HTN				
No		1.00 (Reference)		
Yes	0.950	0.98 (0.52–1.84)		
AChA grade				
0		1.00 (Reference)		
1	0.877	0.83 (0.08–8.45)		
2	0.885	0.84 (0.09–8.36)		
PComA grade				
0–1		1.00 (Reference)		
2	0.848	0.94 (0.51–1.73)		
Suzuki stage, *n*(%)				
I-II		1.00 (Reference)		
III-V	0.171	1.53 (0.83–2.83)		
HDL	0.099	2.03 (0.88–4.72)		
LDL	<0.001	3.43 (2.32–5.08)	0.002	2.46 (1.41–4.28)
TC	<0.001	2.58 (1.86–3.58)	0.104	1.48 (0.92–2.36)
HGB	0.179	0.99 (0.97–1.01)		
TP	0.319	1.02 (0.98–1.07)		
ALB	0.967	1.00 (0.99–1.01)		
TG	0.677	1.11 (0.69–1.77)		

CHD, Coronary heart disease; HGB, Hemoglobin; TP, Total Protein; TG, Triglycerides; TC, Total Cholesterol; HDL, High-Density Lipoprotein; LDL, Low-Density Lipoprotein; AChA grade (anterior choroidal artery grade); PComA grade (posterior communicating artery grade).

**Figure 1:**
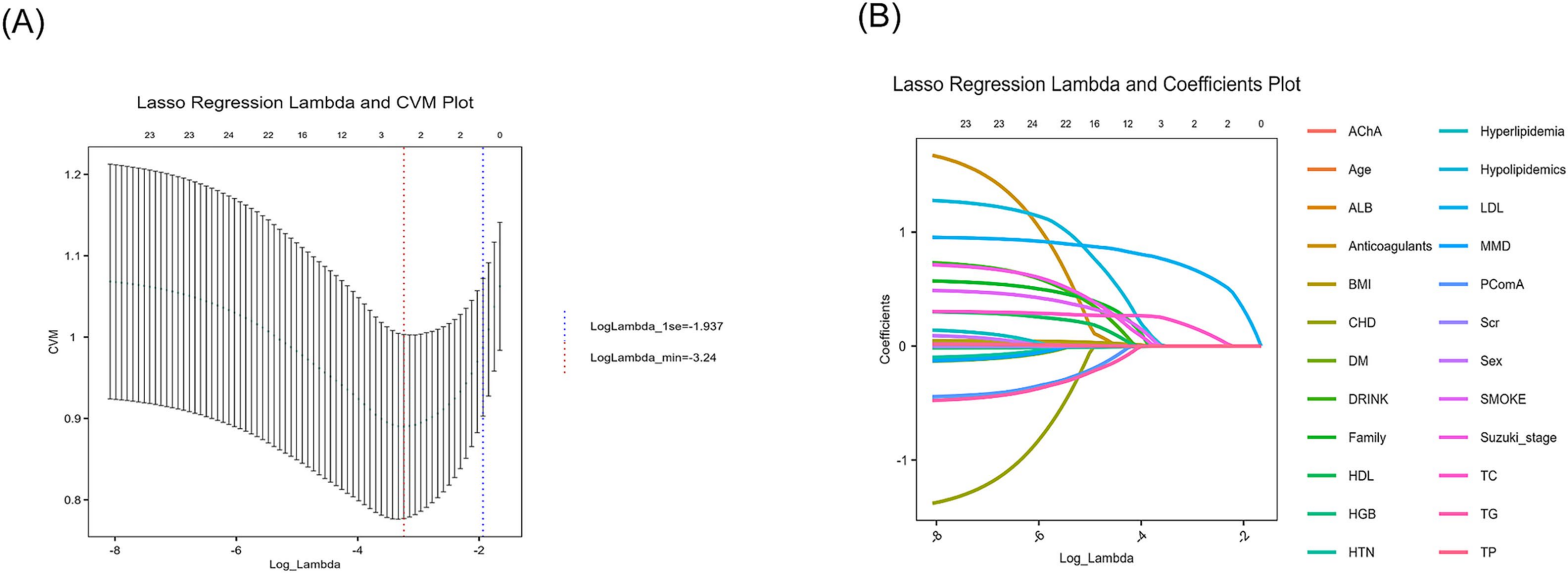
LASSO logistic regression for predictor selection. **(A)** Cross-validation error curve: optimal regularization parameter (*λ*.1se) determined via 10-fold cross-validation to balance performance and parsimony. **(B)** Coefficient trajectory plot: only LDL-C and TC retained non-zero coefficients at λ.1se, identified as core predictors; all other covariates were excluded.

LASSO logistic regression combined with 10-fold cross-validation was performed to screen all candidate predictors of preoperative hemorrhage in adult moyamoya disease patients, effectively mitigating model overfitting and multicollinearity while identifying core predictive factors. Figure 1A displays the cross-validation error curve of the LASSO regression. We selected lambda.1se to balance predictive performance and model simplicity. The numbers at the top of the plot indicate the number of non-zero coefficients retained in the model at different *λ* values. Figure 1B shows the LASSO regression coefficient trajectory plot. As the regularization parameter λ increased, the coefficients of most variables gradually shrank to zero and were eliminated from the model. At the optimal lambda.1se, only LDL-C and TC maintained non-zero regression coefficients, and were thus stably identified as the core predictors of preoperative hemorrhage risk. Other demographic characteristics, imaging grading indicators, and clinical confounding factors were automatically excluded due to weak predictive contribution. Further collinearity diagnosis revealed that the variance inflation factor of LDL-C and TC was 3.017, which was less than 5, suggesting no severe multicollinearity between the two variables and robust parameter estimation results.

LDL-C and TC were subsequently incorporated into the multivariate regression model. Multivariate analysis demonstrated that LDL-C remained an independent risk factor for preoperative hemorrhage (OR = 2.46, 95%CI: 1.41–4.28, *p* = 0.002), whereas TC showed no statistically significant correlation with preoperative hemorrhage (OR = 1.48, 95%CI: 0.92–2.36, *p* = 0.104).

#### 3.2.2 Restricted cubic spline analysis between TC and LDL-C the risk of preoperative bleeding in moyamoya disease

**Figure 2:**
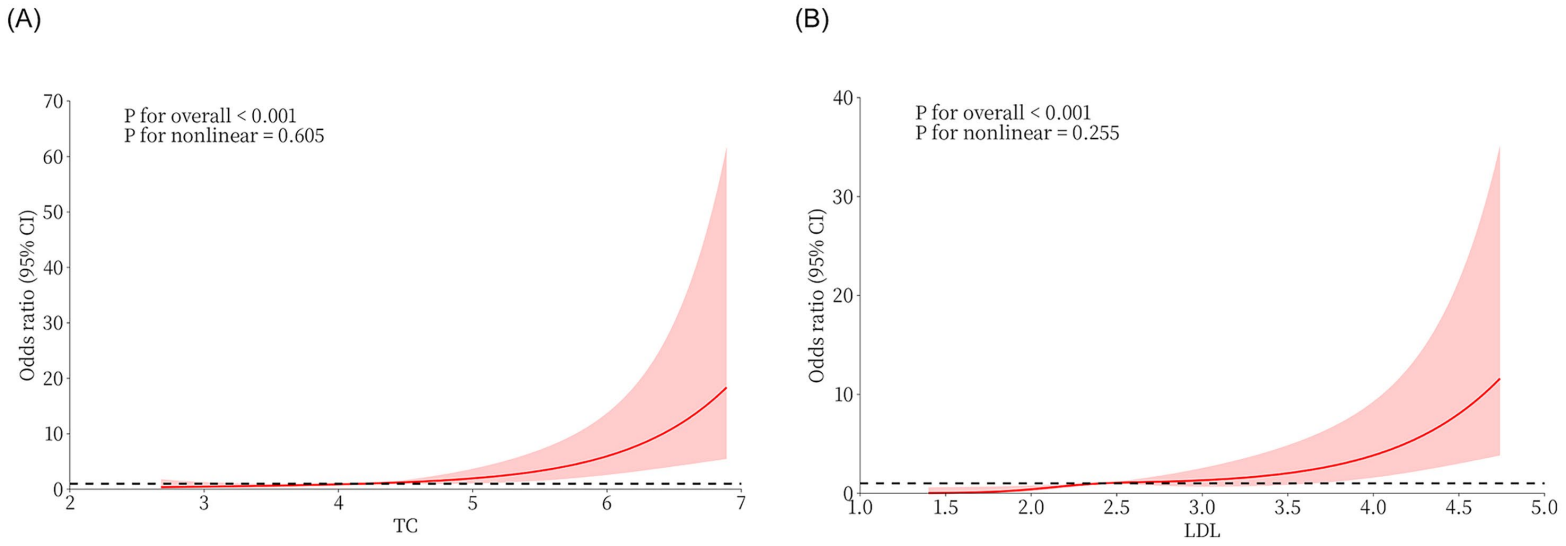
Focusing on the association between TC and LDL-C and preoperative hemorrhage risk in adult moyamoya disease, the linear trend of this relationship was visualized using a restricted cubic spline model.

RCS regression models were applied to systematically analyze the dose–response associations of TC (Figure 2A) and LDL-C (Figure 2B) levels with preoperative hemorrhage risk in MMD patients. Serum TC levels were significantly associated with preoperative hemorrhage risk, and tests for nonlinearity showed no statistically significant deviation from linearity (*p* > 0.05). Although the overall relationship was statistically consistent with linearity, a visual trend of accelerated risk elevation was observed at higher TC concentrations, with the OR of preoperative hemorrhage rising progressively as TC increased. Notably, the OR for preoperative hemorrhage became statistically significant (95% CI entirely above 1) when TC > 5.0 mmol/L, whereas the OR was close to 1 without significant risk elevation in the low TC range. Similarly, serum LDL-C levels were significantly associated with preoperative hemorrhage risk, and no significant nonlinear association was detected (*p* > 0.05). Although the overall association was statistically linear, a visual trend of accelerated risk increase was observed at higher LDL-C concentrations. The fitted curve revealed that the OR of preoperative hemorrhage increased progressively with elevated LDL-C levels, and became statistically significant (95% CI entirely above 1) when LDL-C > 3.5 mmol/L, while no significant risk increase was found in the low LDL-C range.

#### 3.2.3 Construction of a prediction model for preoperative bleeding risk in moyamoya disease

LASSO logistic regression with ten-fold cross-validation identified LDL-C and TC as core predictive factors for preoperative hemorrhage risk in patients with MMD. Given that both indices were selected as key predictors by LASSO regression, TC was retained in the final prediction model to comprehensively reflect the overall lipid metabolic profile and improve the predictive completeness of the model.

**Figure 3:**
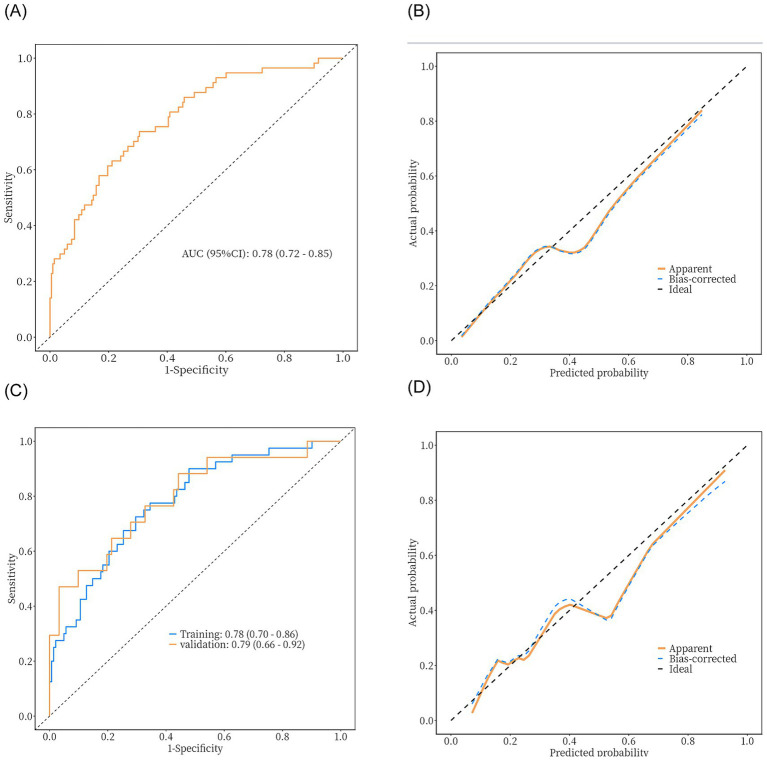
**(A)** Receiver operating characteristic curve and area under the curve value (AUC = 0.78, 95% CI: 0.72–0.85), which was utilized to evaluate the discriminative ability of the prediction model; **(B)** Calibration curve and Hosmer-Lemeshow test result, which was employed to validate the consistency between the predicted probabilities and actual probabilities of the model; **(C)** Comparison of ROC curves between the training set (AUC = 0.78) and validation set (AUC = 0.79), which was designed to assess the generalization performance of the model; **(D)** Calibration curve and Hosmer-Lemeshow test result of the validation set, which was conducted to further confirm the calibration performance of the model.

A preoperative hemorrhage risk prediction model for MMD patients was constructed based on LDL-C and TC. The model performance was initially evaluated using ROC curves and the Hosmer-Lemeshow test. The AUC (Figure 3A) was 0.78 (95%CI: 0.72–0.85), which was higher than the random reference line (AUC = 0.5), indicating that the model had moderate discriminative value for preoperative hemorrhage risk in MMD patients. The lower limit of the 95%CI was greater than 0.5, confirming the statistically significant discriminative efficacy. At the optimal cut-off value of 0.193 determined by the Youden index, the model achieved an accuracy of 0.70 (95%CI: 0.64–0.76), a sensitivity of 0.69 (95%CI: 0.63–0.76), a specificity of 0.74 (95%CI: 0.62–0.85), a positive predictive value (PPV) of 0.90 (95%CI: 0.86–0.95), and a negative predictive value (NPV) of 0.40 (95%CI: 0.31–0.50). These metrics reflected moderate overall classification performance: the high PPV suggests reliable identification of patients with true elevated hemorrhage risk, while the relatively lower NPV aligns with the clinical challenge of ruling out low-risk cases in this setting. In the Hosmer-Lemeshow test (Figure 3B), both the apparent calibration curve and the bootstrap bias-corrected curve fitted well with the ideal 45° reference line, suggesting favorable internal validation and acceptable predictive performance of the model, with no obvious overfitting and stable calibration between the predicted probability and the actual hemorrhagic event.

Subsequently, all participants were randomly divided into a training set and a validation set at a 7:3 ratio to further verify the model’s discriminative performance and calibration. ROC curve analysis revealed that the AUC (Figure 3C) was 0.78 (95%CI: 0.70–0.86) in the training set and 0.79 (95%CI: 0.66–0.92) in the validation set, both of which were above the random reference line with comparable AUC values and confidence intervals. These results indicated moderate discriminative value in the training cohort and stable and acceptable predictive performance in the independent validation cohort, with no significant overfitting. The Hosmer–Lemeshow test (Figure 3D) demonstrated that both the apparent calibration curve and the bootstrap-corrected curve approximated the ideal 45° reference line, with only slight fluctuations in the medium–high probability interval, suggesting favorable internal validation and good consistency between model-predicted probabilities and actual hemorrhage events.

In conclusion, the preoperative hemorrhage risk prediction model for MMD patients based on LDL-C and TC demonstrates moderate discriminative value and favorable calibration in both the training and validation sets, with stable and acceptable predictive performance. This model may provide a useful reference for preoperative hemorrhage risk stratification and individualized clinical decision-making in patients with MMD.

#### 3.2.4 Construction of a preoperative bleeding risk nomogram for patients with MMD based on LDL-C and TC and verification of its clinical efficacy

**Figure 4:**
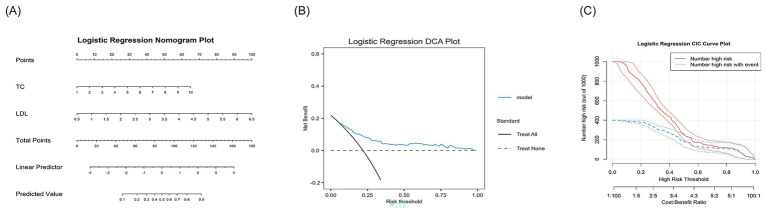
**(A)** Nomogram, which visualizes the prediction model to facilitate rapid clinical quantification of preoperative hemorrhage risk; **(B)** Decision curve analysis plot, which evaluates the clinical net benefit of the model across diverse risk thresholds, providing actionable insights for clinical intervention decision-making; **(C)** Clinical impact curve of the preoperative bleeding risk prediction model for patients with Moyamoya disease.

A preoperative hemorrhage risk prediction model was established for MMD patients based on serum LDL-C and TC. A nomogram was plotted to achieve individualized visual risk assessment, and its clinical application value was systematically verified using DCA and CIC. The nomogram (Figure 4A) incorporated LDL-C and TC as predictors; higher lipid levels corresponded to higher individual scores, and the total score directly reflected the probability of preoperative hemorrhage, providing a convenient visual tool for clinical preoperative risk stratification.

DCA (Figure 4B) showed that across a broad range of risk thresholds (approximately 5–85%), the net benefit of the model was higher than that of the “Treat All” and “Treat None” strategies. This suggests that within clinically relevant risk thresholds, model-based risk assessment could provide incremental net benefit for identifying patients at high preoperative hemorrhage risk, while helping to reduce excessive medical interventions, indicating acceptable clinical applicability and moderate decision-supporting value of the model.

CIC further confirmed (Figure 4C) that within the commonly used clinical cost–benefit ratio range of 1:5 to 5:1, the model stably identified patients at high risk of preoperative hemorrhage, with a consistently high proportion of events in the high-risk cohort. As the risk threshold increased, the number of high-risk patients decreased gradually, but the event rate in the high-risk group remained relatively stable, effectively balancing intervention benefits and the risk of over-treatment.

In conclusion, the preoperative hemorrhage risk prediction nomogram for MMD based on LDL-C and TC is simple to operate and highly visual. Verified by DCA and CIC, it presents stable clinical net benefit and favorable clinical practicability, providing a reference for individualized preoperative hemorrhage risk assessment and clinical decision-making in MMD patients.

## 4 Discussion

The present study identified three core and clinically relevant findings for preoperative hemorrhage in adult MMD. First, elevated LDL-C was confirmed as an independent risk factor for preoperative intracranial hemorrhage (OR = 2.46, 95%CI: 1.41–4.28, *p* = 0.002), and both LDL-C and TC showed an approximately linear positive correlation with hemorrhagic risk, with approximate cutoff values of 3.5 mmol/L and 5.0 mmol/L, respectively. Second, the prediction model based on LDL-C and TC showed moderate discriminative value (AUC = 0.78, 95%CI: 0.72–0.85) and acceptable diagnostic performance (accuracy = 0.70, sensitivity = 0.69, specificity = 0.74, positive predictive value = 0.90), with stable performance in training and internal validation sets and no obvious overfitting; however, this model was developed in a sample of only 260 patients and lacks external validation. Third, the constructed nomogram showed acceptable clinical net benefit across a range of risk thresholds and may serve as a preliminary simple visual tool for individualized preoperative hemorrhage risk stratification.

Currently, the focus of research in the field of MMD lies predominantly on postoperative cerebral ischemic complications, whereas analyses regarding the risk factors for preoperative intracerebral hemorrhage events remain relatively scarce. Preoperative hemorrhage in MMD entails extremely detrimental consequences. The mass effect of the intracranial hematoma resulting from such hemorrhage can precipitate a sharp elevation in intracranial pressure, which in turn leads to the compression and displacement of brain tissue and the disruption of the normal anatomical structure. Consequently, formidable challenges arise in aspects like the selection of surgical approaches, the exposure of diseased blood vessels, and the precision of surgical manipulations (19, 20). Moreover, preoperative hemorrhage serves to exacerbate the cerebral ischemic state. Given that the vascular stenosis or occlusion induced by MMD has already given rise to insufficient cerebral perfusion, the exacerbation of local blood circulation disorders following hemorrhage triggers more extensive ischemic and hypoxic damage to brain tissue (21, 22). Simultaneously, hemorrhage and the subsequent decomposition products of the hematoma can stimulate the surrounding brain tissue, thereby instigating inflammatory responses that culminate in increased damage and apoptosis of nerve cells and ultimately impede the recovery of nerve functions. As a result, patients may manifest more severe clinical symptoms, including limb movement disorders, speech function impairments, and cognitive decline. Furthermore, as the disease progresses, these neurological deficits are prone to persistent aggravation and are often refractory to reversal (23–25). Against this backdrop, our study performed a statistical analysis of the risk factors for preoperative hemorrhage in patients with MMD by collecting common biochemical indicators as well as basic clinical data. As a retrospective observational study, our findings only identified an observational association that lower LDL-C levels were correlated with a reduced risk of preoperative intracerebral hemorrhage, rather than confirming a causal protective effect. Based on these results, we established a relevant predictive model, with the aim of furnishing valuable references for clinical practice and facilitating the optimization of preoperative risk assessment and management strategies.

Given that this is a retrospective observational study, we can only demonstrate statistical associations between LDL-C/TC levels and preoperative hemorrhagic risk in MMD patients, rather than verifying a causal relationship. No interventional evidence is available in this study, and the following pathophysiological discussions are all theoretical inferences based on existing literature, aiming to explain the observed statistical correlation, which cannot be regarded as proof of causality. Based on literature review, this study investigated the possible pathophysiological mechanisms underlying the observed association between elevated LDL-C, TC and preoperative hemorrhage in patients with MMD. These lipid abnormalities target the thin and fragile abnormal neovessels at the skull base in MMD patients, causing superimposed injuries via endothelial dysfunction, structural degradation, hemodynamic disturbance and other dimensions, thereby significantly lowering the vascular rupture threshold. The possible mechanism is as follows:1. Severe endothelial damage and exhausted vasodilatory capacity: High TC strongly inhibits endothelial nitric oxide synthase (eNOS) activity and the downstream eNOS-cGMP-PKG signaling cascade, directly impairing endothelial vasodilatory function (26, 27); Meanwhile, extensive oxidation of LDL-C to ox-LDL directly injures the fragile vascular endothelium in MMD, disrupts endothelial barrier integrity, and induces endothelial cell apoptosis and shedding (28, 29). Such dual damage greatly weakens the buffering capacity of compensatory neovessels against blood pressure fluctuations, disturbs vascular wall stress distribution, and substantially reduces the vascular anti-rupture threshold. 2. Vascular wall matrix degradation and compromised structural stability: ox-LDL significantly upregulates the expression of matrix metalloproteinases (MMPs, such as MMP-9) and directly degrades key extracellular matrix components including collagen and elastin in the vascular wall (30, 31); Elevated total cholesterol may exacerbate endothelial injury and lipid deposition in the distal internal carotid artery, which underlies the progressive occlusive vasculopathy in moyamoya disease (32–34). The aberrant moyamoya collaterals inherently lack smooth muscle and elastic fiber support; such lipid-related endothelial damage further compromises vascular wall integrity, markedly reducing the rupture threshold of these fragile vessels under intracranial hemodynamic stress.3. Amplified chronic inflammatory cascade and vascular wall erosion: High TC activates the monocyte/macrophage system, induces ox-LDL formation and releases abundant pro-inflammatory factors (TNF-*α*, IL-6, CRP, etc.), initiating and perpetuating chronic vascular inflammation (35); Meanwhile, LDL-C directly activates inflammatory signaling pathways in the vascular wall; sustained infiltration of inflammatory cells erodes the fragile moyamoya vessel wall and generates local weak spots, which act as critical breakthroughs leading to hemorrhage (36). 4. Hemodynamic disturbances and abnormal shear stress injury: Elevated TC increases whole-blood and plasma viscosity and reduces blood flow velocity, resulting in microcirculatory stasis (37–39); Increased LDL-C also elevates blood viscosity, worsening the already impaired cerebral perfusion in patients with MMD (40). Diminished flow and vortex formation cause aberrant shear stress distribution and heightened cyclic loading, which continuously traumatize the compromised fragile vascular wall and substantially raise rupture risk.5. Coagulation–fibrinolysis imbalance and abrupt elevation of intravascular pressure: LDL-C triggers excessive platelet aggregation, favoring microthrombosis and consequent focal luminal stenosis with sharp increases in intraluminal pressure (41). It also impairs fibrinolytic activity, exacerbating blood stasis and further elevating intravascular pressure. These effects ultimately exceed the tensile limit of fragile MMD vessels and precipitate hemorrhagic rupture. In summary, this study tentatively integrates the potential pathophysiological mechanisms of elevated LDL-C and TC associated with intracranial hemorrhage in MMD based on existing literature. It should be noted that this mechanism is derived from literature inference, lacking direct experimental validation, and specific molecular details remain unclear. Conclusions should therefore be interpreted cautiously, with future studies needed to verify and refine this framework.

This study has several limitations that warrant cautious interpretation of the results. First and foremost, this is a retrospective observational study, which can only confirm statistical associations but cannot establish a causal relationship between LDL-C/TC levels and preoperative hemorrhagic risk. There is a lack of interventional data, and the present findings are insufficient to support any clinical conclusions that modifying LDL-C/TC levels can alter the hemorrhagic risk of MMD patients. Second, only admission lipid levels were included, without analysis of perioperative dynamic lipid changes, which may overlook the impact of lipid fluctuations on hemorrhagic risk. In addition, detailed data on lipid-lowering therapy were not collected, preventing verification of the causal relationship between lipid reduction and decreased hemorrhagic risk. These limitations do not affect the reliability of the core results and provide directions for future research. Future investigations on this topic may also explore *in vitro* and *in vivo* experiments to verify the molecular mechanisms underlying lipid-induced vascular injury in MMD, incorporate dynamic perioperative lipid monitoring and neuroimaging markers to optimize the predictive model, and conduct long-term follow-up studies to clarify the association between lipid profiles and postoperative rebleeding and long-term neurological prognosis in MMD patients.

In conclusion, elevated LDL-C may be an independent risk factor for preoperative intracranial hemorrhage in adult MMD patients, and both LDL-C and TC levels show linear positive correlations with preoperative hemorrhage risk. The prediction model and nomogram constructed based on LDL-C and TC exhibit favorable discrimination, calibration and clinical practicability, and may be applied for rapid preoperative risk stratification in adult MMD patients. These findings suggest that perioperative lipid monitoring and management may be of great significance in the clinical management of MMD, potentially providing a simple and practical tool for clinical risk assessment and decision-making assistance. Further multicenter prospective studies are warranted to validate the external validity of this model and explore the actual value of lipid-lowering interventions in reducing preoperative hemorrhagic events in adult MMD patients.

## Data Availability

The raw data supporting the conclusions of this article will be made available by the authors, without undue reservation.
